# The Pattern of CT Scan Use in the Diagnosis of Abdominal Pain in Children Presenting to the Emergency Department of a Tertiary Community Hospital

**DOI:** 10.7759/cureus.19162

**Published:** 2021-10-31

**Authors:** Hamza H Khan, Shova Subedi, Sanjay Kumar, Hernando Lyons

**Affiliations:** 1 Medicine, Shifa International Hospital, Islamabad, PAK; 2 Pediatrics, Ascension St. John Hospital, Detroit, USA; 3 Pediatric Gastroenterology, Medical University of South Carolina, Charleston, USA; 4 Pediatric Gastroenterology, Brown University, Providence, USA; 5 Pediatric Gastroenterology, Ascension St. John Hospital, Detroit, USA

**Keywords:** appendicitis, emergency department, ct scan, pediatrics, abdominal pain

## Abstract

Background and objective

Pediatric populations are highly sensitive to ionizing radiations and, therefore, are more at risk of their harmful outcomes. Our study aimed to determine the percentage of children who underwent a CT scan after presenting to the ED with abdominal pain. The secondary aim was to determine the change in management related to the CT results. In addition, we also wanted to determine the predictors associated with the use of abdominal CT scans in the evaluation of children presenting to ED with abdominal pain as well as the predictors associated with positive CT scan results in children with abdominal pain.

Materials and methods

We retrospectively reviewed the medical records of children with abdominal pain seen in our ED from 01/01/2011 through 12/30/2012. Patients aged 4-18 years presenting with abdominal pain were identified from the medical records. Data on demographics, clinical characteristics, associated factors, CT use, CT findings, and change in management were collected. Data were analyzed using Chi-square (χ^2^) analysis and Student’s t-test.

Results

A total of 1,780 charts were reviewed and 1,272 children were included in the study. The mean age of the cohort was 12.6 ± 4.6 years; 62.6% were female and 68.7% were African American. Of note, 14% (181/1,272) of the study group had received a CT scan; change in medical management was noted in 34.8% (63/181) of the scanned patients. Predictors of CT use included older age (p<0.0001), male gender (p<0.0001), white race (p<0.0001), an attending without advanced training in pediatric emergencies (p=0.001), acute onset of symptoms (p<0.0001), higher pain score (p<0.0001), right lower quadrant pain (p<0.0001), abdominal wall rebound tenderness (p<0.0001), abdominal tenderness (p<0.0001), fever (p<0.0001), and absence of constipation (p=0.04). Positive CT scan results were predicted by the presence of fever (p=0.013), lack of constipation (p=0.025), and white race (p=0.022). A multivariate analysis could not be done because not all data were available for each patient.

Conclusion

The use of the CT scan in children with abdominal pain affected the management in one out of three patients (34.8%). Fever, constipation, and white race were the factors associated with an increased likelihood of performing a CT scan and were also linked to positive results.

## Introduction

Exposure to Ionizing radiation through diagnostic studies has become a serious public health concern. The largest source (up to 60%) of iatrogenic exposure to ionizing radiation is CT scans [1-3]. One-third of CT scans (≈1 million annually) are done unnecessarily [1]. Abdominal CT scans are potentially more dangerous as they require a higher dose of radiation and the abdominal organs are more sensitive to radiation-induced cancer [4]. This is of particular concern in the pediatric population because their organs have increased sensitivity to radiation and the radiation-induced damage is expressed for a longer period compared to the adult population [5,6].

The use of CT scans has increased substantially, particularly in the ED [7,8]. In Adult ED, from 1995 to 2007, 2.7-16.2 million CT scans were performed [9]. This reflects a 5.9 fold increase with a compound annual growth rate of 16% [9]. On the other hand, in pediatric ED, 0.33-1.65 million CT scans were done from 1995 to 2008, which shows a five-fold increase and a compound annual growth rate of 13.2% [10]. However, in their study, Chang et al. report a decreasing trend in abdominal CT use in children since 2007 [4].

In light of the increased susceptibility of the pediatric population to potential harmful outcomes of ionizing radiation, our study aimed to determine the percentage of children who underwent a CT scan after presenting to the ED with abdominal pain. The secondary aim was to determine the change in management related to the CT results. Additionally, we sought to determine the predictors associated with the use of abdominal CT scans in the evaluation of children presenting to ED with abdominal pain as well as the predictors associated with positive CT scan results in children with abdominal pain.

## Materials and methods

In this retrospective review study, we examined the medical records of children seen in the ED at St. John Hospital and Medical Center (SJHMC) with complaints of abdominal pain between January 2011 to December 2012. SJHMC is a community teaching hospital and the ED at SJHMC has a dedicated 15-room pediatric section that focuses entirely on the needs of the injured and ill children, with a patient turnout of over 28,000 every year. The facility has five pediatric emergency specialists with occasional coverage provided by general emergency specialists. In addition, one of the two pediatric surgeons is always on call to address surgical issues, but ordering a CT scan does not require permission from the surgeon. Patients have access to 24-hour diagnostic radiological workups including a bedside ultrasound facility.

The study subjects were identified per the diagnosis at the time of ED registration. Our inclusion criteria included pediatric patients of both genders and all ethnic groups between the ages of 4-18 years presenting with a chief complaint of abdominal pain, and patients seen by both pediatric emergency and general emergency specialists. The rationale for choosing this age group was that patients younger than four years of age cannot express themselves adequately and patients older than 18 years of age are more often seen in the adult ED. We excluded patients with an established diagnosis of inflammatory bowel disease, celiac disease, kidney stones, gall-bladder stones, eosinophilic gastroenteritis, and patients with a history of abdominal trauma and/or abdominal surgery in the previous month. The aim of excluding these established diagnoses was to limit the confounding factors in our study population.

The data that we collected included patients’ demographic features (age, gender, race), clinical manifestations, pain characteristics, and associated symptoms, type of emergency room physician, CT scan findings, and change in medical or surgical treatment due to imaging findings.

The data were analyzed using χ2 analysis, the Mann-Whitney U test, and Student’s t-test. All analyses were performed using SPSS Statistics v.21.0 (IBM, Armonk, NY), and a p-value of 0.05 was considered statistically significant. The study was approved by the SJHMC Institutional Review Board (IRB).

## Results

**Figure 1:**
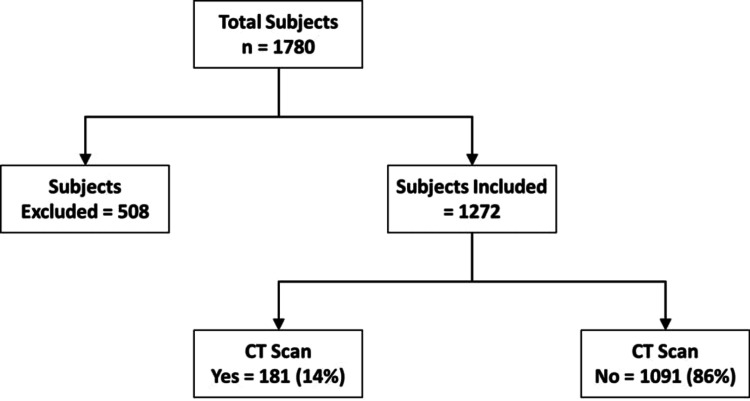
Consort diagram representing the breakdown of exclusions to reach the final number of included patients CT: computed tomography

We reviewed a total of 1,780 charts and based on our exclusion criteria, we excluded 508 charts, which narrowed down our study population to 1,272 (Figure 1).

**Figure 2:**
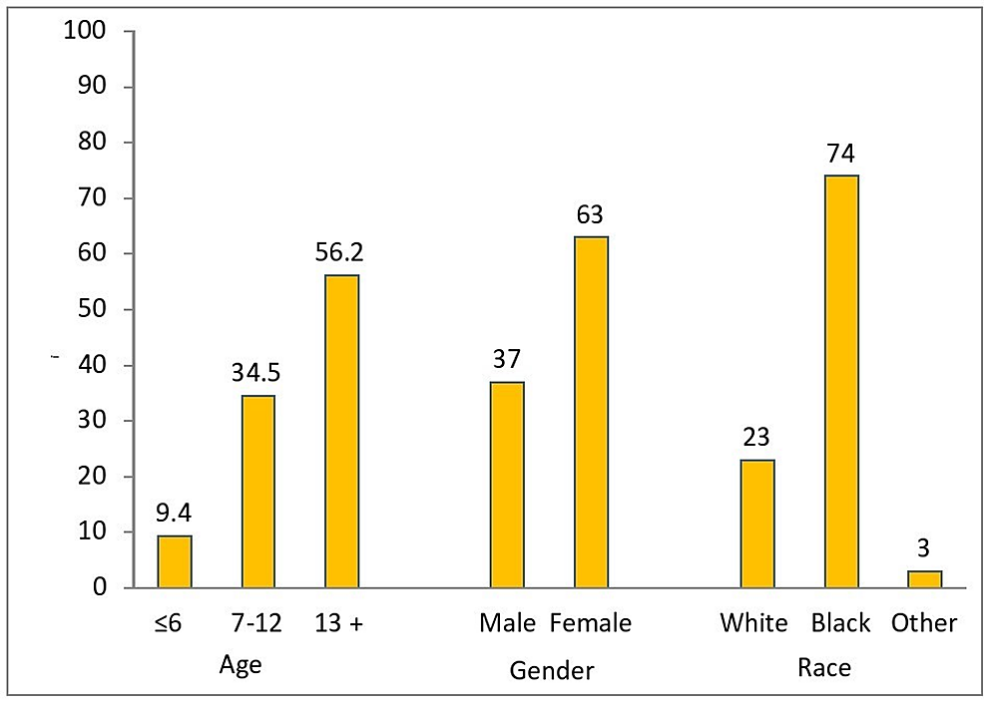
Demographic characteristics (in percentages) based on age, gender, and race

Figure 2 outlines the demographic details of our patients. The mean age of the cohort was 12.6 ± 4.6 years.

**Figure 3:**
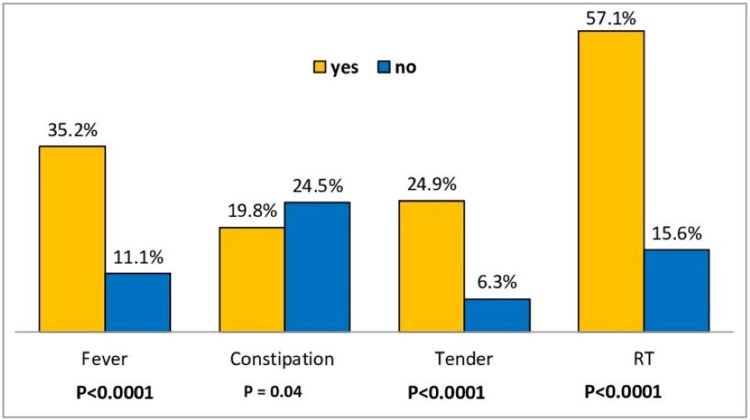
Predictors of CT scan use based on fever, constipation, abdominal tenderness, and rebound tenderness CT: computed tomography; RT: rebound tenderness

Of note, 14% (n=181) of the study group received a CT scan. The predictors of CT use included older age (p<0.0001), male gender (p<0.0001), white race (p<0.0001), an attending without advanced training in pediatric emergencies (p=0.001), acute onset of symptoms (p<0.0001), higher pain score (p<0.0001), right lower quadrant pain (p<0.0001), abdominal wall rebound tenderness (p<0.0001), abdominal tenderness (p<0.0001), fever (p<0.0001), and absence of constipation (p=0.04). Other variables for the prediction of CT use with respective p-values were as follows: bloody stools (p=0.34), diarrhea (p=0.42), nausea (p=0.11), vomiting (p=0.83), nature of pain (p=0.27), and rigidity (p=0.89). Tables 1, 2, and Figure 3 summarize the predictors of CT scan use.

**Table 1:** Predictors of CT scan use based on age, gender, race, emergency department physician, location of pain, and severity of pain G: generalized; UA: upper abdomen; LoA: lower abdomen; RA: right abdomen (right flank); LA: left abdomen (left flank); M: midline; RLQ: right lower quadrant; CT: computed tomography

Variable		Patients with data, n (%)	CT done	No CT	P-value
Mean age in years [patients with data, n (%): 1,265 (99.2)]			14.9 ± 3.4	12.2 ± 4.7	<0.0001
Gender	Male	1,265 (99.2)	49.70%	35.50%	<0.0001
	Female		50.30%	64.50%	
Race	African American	1,188 (93)	59.6%	75.5%	<0.0001
	Caucasian		37.30%	21.80%	
	Others		3.00%	2.60%	
Physician	Adult	1,099 (86)	35.4%	22.5%	0.001
	Pediatric		64.60%	77.50%	
Pain location	UA	1,188 (93)	0.6%	5.4%	<0.0001
	G		16.00%	38.90%	
	M		8.60%	13.10%	
	LA		6.20%	8.80%	
	LoA		19.10%	18.80%	
	RA		16.70%	6.30%	
	RLQ		32.70%	8.70%	
Onset of symptoms	Acute	509 (40)	76.5%	53.8%	<0.0001
	Gradual		23.50%	46.20%	
Mean pain severity		862 (68)	8.13 ± 1.97	7.06 ± 2.19	<0.0001

**Table 2:** Predictors of CT scan use based on symptoms; bloody stool, diarrhea, nausea, vomiting, nature of pain, and abdominal rigidity CT: computed tomography

Variable		CT done	No CT	P-value
Bloody stool		5.7%	11.2%	0.34
Diarrhea		51.1%	57.7%	0.42
Nausea		94.0%	98.0%	0.11
Vomiting		77.2%	76.2%	0.83
Nature of pain	Aching	24.1%	23.6%	0.27
	Burning	2.5%	6.7%	
	Cramping	17.7%	23.9%	
	Sharp	53.2%	41.8%	
	All other	2.5%	4.0%	
Rigidity		1.0%	1.1%	0.89

**Figure 4:**
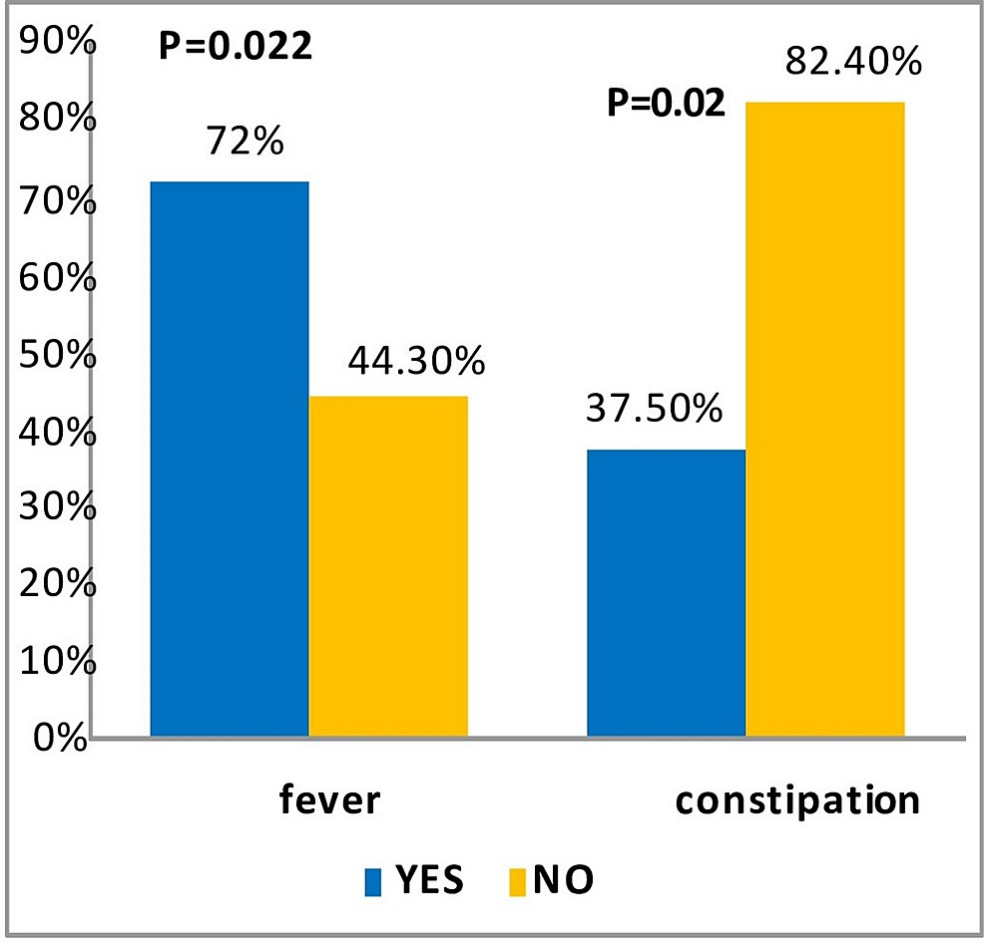
Predictors of positive results in the CT scan with respect to fever and constipation CT: computed tomography

Positive CT scan results were predicted by the presence of fever (p=0.013), lack of constipation (p=0.025), and white race (p=0.022) (Tables 3, 4, and Figure 4).

**Table 3:** Predictors of positive results in the CT scan based on the race of the patient (African American, Caucasian, others) and the presence of fever CT: computed tomography

Variable	Patients with data, n (%)	Positive CT	Negative CT	P-value
Race	164 (90%)			
African American		50.0%	70.5%	0.022
Caucasian		45.3%	28.2%	
Others		4.7%	1.3%	
Fever		27.7%	10.6%	0.013

**Table 4:** Predictors of positive results in the CT scan based on age, gender, and duration and nature of symptoms CT: computed tomography

Variable	Positive CT	Negative CT	P-value
Mean age in years	14.6 ± 3.4	15.1 ± 3.5	0.31
Male; female	51.1%; 48.9%	48.3%; 51.7%	0.71
Duration of pain, days, median (range)	2.0 (0-60)	2.0 (0-365)	0.12
Acute onset; gradual onset	74.1%; 25.9%	79.1%; 20.9%	0.57
Rebound tenderness	12.8%	8.3%	0.53
Diarrhea	50.0%	52.4%	0.87
Nausea	94.1%	93.8%	0.95
Vomiting	77.6%	76.1%	0.85

Rest of the variables related to positive CT scan results were as follows: age (p=0.31), gender (p=0.71), duration of pain (p=0.12), onset of symptoms (p=0.57), rebound tenderness (p=0.53), diarrhea (p=0.87), nausea (p=0.95), and vomiting (p=0.85). Table 5 summarizes the CT scan diagnosis of the patients.

**Table 5:** Summary of the CT scan diagnosis of the patients CT: computed tomography; UTI: urinary tract infection; GI: gastrointestinal

Diagnosis	Frequency
Appendicitis	47
Unspecified abdominal pain	45
Renal stones	16
Gastroenteritis	15
Ovarian cyst	13
Constipation	12
Mesenteric adenitis	9
Other gynecological problems	5
UTI	3
Abscess	3
Biliary stone/colic	2
GI bleed	1
Others	10

The CT scan was reported to be normal in 26% (n=47), abnormal in 55.8% (n=101), and non-specific in 18.2% (n=33) of patients. The results of the CT scan changed medical management in 34.8% (n=63/181) of the scanned patients (p<0.0001). Given the retrospective nature of the study, not all data was available for all patients; therefore, the multivariate analysis and logistic regression to examine the potential variables for a positive CT scan could not be done.

## Discussion

Even though the pediatric population is more prone to damage from ionizing radiations compared to the adult population, a steady upward trend was noted in the western world regarding the use of CT scans in particular for children in the ED until the year 2008 [4,9,11]. Factors responsible for this increasing trend included the benefit of efficiency, time, and cost secondary to improved diagnosis, the less invasive nature of this imaging modality, malpractice litigation concerns, increasing availability of CT scans, and improvement in image resolution since the introduction of multidetector CT (MDCT) [9-14].

However, recent literature reports that CT scan utilization has decreased since 2008 [15]. The use of the Pediatric Emergency Care Applied Research Network (PECARN) for head injury and Alvarado score for abdominal pain and the introduction of other imaging modalities including the “ultrasound first” approach have contributed to this decrease in the use of the CT scan [16].

This increased exposure to ionizing radiation early on in life predisposes pediatric patients to the development of cancers. In addition, pediatric patients end up getting a 50% higher dose of radiation as compared to an adult because of their smaller body size. Also, there are a larger number of cells actively going through the process of cell division, which are more prone to ionizing radiation-related damage, resulting in a longer lead time to develop cancers [5,17]. According to Brenner et al., the possibility of a one-year-old developing cancer is 10-15 times higher compared to a 50-year-old adult exposed to the same dose of radiation [5]. In addition, the increased possibility of pediatric patients undergoing more than one CT scan increases the cumulative radiation exposure and thus the risk of developing cancers. Furthermore, the predicted number of years of life left for a one-year-old child is much higher than it is for a 50-year-old adult, resulting in children having more time to develop complications secondary to their exposure to the radiation. In our study, we found that as much as 14% (n=181) of the study population had a CT scan for abdominal pain. Of note, these figures exclude patients with established diagnoses such as inflammatory bowel disease, celiac disease, kidney stones, gall-bladder stones, eosinophilic gastroenteritis, and patients with a history of abdominal trauma and/or abdominal surgery in the preceding month.

On average, a CT scan costs $898 in the US, and the mean number of CT scans performed in the US per 1,000 population is 245 compared to 151 in other countries [17]. This modality of imaging has no doubt significantly contributed to the health-related economic burden globally, more so in the western world given its excessive use as compared to the rest of the world. So, judicious use of CT scans will definitely reduce health delivery costs and reduce iatrogenic radiation-induced cancers.

While the current trend of CT use and the predictive factors are well documented in the literature, our study adds to the existing literature by highlighting the predictive factors unique to the pediatric population. This study has several limitations. Firstly, this was a single-center study and the study sample was not large enough to reflect general US hospitals trends. The generalizability of our findings is also limited by the fact that this study was conducted in a teaching hospital where resident-ordering effects come into play and hence the results can only be applied in this specific context. Secondly, our study included otherwise healthy patients with first-time exposure to CT scans, and hence the data does not reflect cumulative radiation exposure in patients with underlying diagnosed medical conditions that get repeatedly imaged. Lastly, this was a retrospective study and, while reviewing the charts, we discovered that all variables related to the patient population were not documented. Therefore, multivariate analysis and logistic regression to examine the potential variables for a positive CT scan could not be done. We recommend that a multiyear and multicenter research study be conducted in the future, which would generate results generalizable to a wider population.

## Conclusions

In our study, a CT scan was performed for 14% of pediatric patients presenting to the ED with abdominal pain. The use of CT scans in abdominal pain affected the management in a substantial proportion (34%) of the children. Some of the factors associated with the increased likelihood of performing a CT scan are also associated with positive CT scan findings; these factors include the Caucasian race, the presence of fever, and no history of constipation. Our findings are unique and relevant to emergency physicians, regarding the care of pediatric patients who present to the ED with abdominal pain. In addition to exposure to harmful radiations, CT scans pose a huge burden on the healthcare and economy of a country. Therefore, this study provides useful information regarding CT scan use and how we can avoid unnecessary radiation exposure and treatments by focusing instead on measures such as thorough history and clinical examination, thereby reducing unnecessary healthcare and economic burden on a country.
